# Dieulafoy’s Lesion of the Duodenum: A Rare and Fatal Cause of Gastrointestinal Bleed

**DOI:** 10.7759/cureus.40050

**Published:** 2023-06-06

**Authors:** Abeer Qasim, Patrik Schmidt, Tanushree Bhatt, Vikram Itare, Ariyo Ihimoyan, Misbahuddin Khaja, Sameer Kandhi

**Affiliations:** 1 Internal Medicine, BronxCare Health System, Bronx, USA; 2 Gastroenterology, BronxCare Health System, Bronx, USA

**Keywords:** acute gastrointestinal bleeding, extragastric location of dieulafoy's lesion, duodenal dieulafoy’s disease, dieulafoy’s ulcer, extragastric dieulafoy's lesion

## Abstract

Dieulafoy’s lesion (DL) is an unusual cause of recurrent gastrointestinal bleeding that can be fatal. It can occur in various parts of the gastrointestinal (GI) tract, most commonly located in the stomach, especially at the level of lesser curvature; however, it can occur in other parts, including the colon, esophagus, and duodenum. A duodenal Dieulafoy lesion is characterized by the presence of a larger-caliber artery that protrudes through the GI mucosa and can lead to massive hemorrhage. The exact cause of DL is yet to be determined. Clinical presentation includes painless upper GI bleeding, including melena, hematochezia, and hematemesis, or rarely iron deficiency anemia (IDA); however, most of the patients are asymptomatic. Some patients also have non-gastrointestinal comorbidities such as hypertension, diabetes, and chronic kidney disease (CKD). The diagnosis is established by esophagogastroduodenoscopy (EGD), which includes the presence of micro pulsatile streaming from a mucosal defect, the appearance of a fresh, densely adherent clot with a narrow point of attachment to a minute mucosal defect, and the visualization of a protruding vessel with or without bleeding. Initial EGD can be non-diagnostic due to the relatively small size of the lesion. Other diagnostic modalities include endoscopic ultrasound and mesenteric angiography. The treatment of duodenal DL includes thermal electrocoagulation, local epinephrine injection, sclerotherapy, banding, and hemoclipping. We present here a case of a 71-year-old female who had a history of severe IDA requiring multiple blood transfusions and intravenous iron in the past and was found to have duodenal DL.

## Introduction

A Dieulafoy lesion (exulceratio simplex, cirsoid aneurysm, or caliber-persistent submucosal vessel) is a cause of gastrointestinal bleeding (GI), which is characterized by a small artery with an abnormally dilated caliber that protrudes through the mucosal surface of the gastrointestinal tract. It is rare and can be life-threatening. The ulcerative lesion in patients with duodenal dieulafoy lesion (DL) is located around 6-10cm from the gastroduodenal junction. Most commonly, DL is located in the stomach but can be found in the duodenum rarely, mostly at the level of the duodenal bulb (53%), followed by the third part of the duodenum (29%), and at the junction of the first and second parts of the jejunum (18%) [1]. Duodenal DL contributes to 15% of all DL lesions and constitutes 3.5% of all GI bleeding. The mortality ranges between 23% and 79% [2]. The diagnosis of duodenal DL can be challenging as most patients are asymptomatic or present with massive bleeding without any signs or symptoms. Early endoscopic evaluation can be helpful for definitive diagnosis as well as treatment [3]. In this case report, we present a patient with duodenal DL who was managed successfully via endoscopic intervention.

## Case presentation

A 71-year-old female with a past medical history of asthma, diabetes mellitus, peripheral vascular disease, hypertension, depression, anxiety, osteomyelitis of bilateral feet, substance abuse disorder, chronic insomnia, ischemic colitis, hemorrhoids, tubular adenomatous polyp, and pulmonary hypertension presented to the emergency room with complaints of shortness of breath, malaise, and abdominal pain for the past several weeks. She had a surgical history of amputation of the first, second, and third toes of the left foot and a transverse colectomy with diverting ileostomy that was later reversed. She denied any toxic habits or drug allergies. The patient has a history of severe anemia requiring multiple blood transfusions and intravenous iron infusions. Her vitals on admission showed a temperature of 97.3F, a pulse of 76 beats per minute, a blood pressure of 115/57 mmHg, and an oxygen saturation of 97% in room air. A physical exam revealed bibasilar crackles and right upper quadrant abdominal tenderness. Laboratory investigations on admission were significant for anemia with a hemoglobin of 6.7 g/dl and hematocrit of 19.6% and thrombocytosis with a platelet count of 417K/ul (Table 1).

**Table 1:** Laboratory findings on admission.

Laboratory parameters	Results	Reference range and units
Hemoglobin	6.7 g/dL	12–16 g/dL
Hematocrit	19.6%	42-51%
White blood cell count	5.8 K/µL	4.8–10.8 K/µL
Platelets	417 K/µL	150–400 K/µL
Sodium	137 mEq/L	135–145 mEq/L
Potassium	4.6 mEq/L	3.5–5.0 mEq/L
Blood urea nitrogen	25 mg/dL	6–20 mg/dL
Creatinine	1 mg/dL	0.5–1.5 mg/dL
Alanine aminotransferase	20 units/L	5-40 units/L
Aspartate aminotransferase	42 units/L	9-33 units/L
Alkaline phosphatase	104 units/L	43-160 units/L
Iron	6 µg/dL	65-175 µg/dL
Unsaturated iron binding capacity	173 µg/dL	112-346 µg/dL
Transferrin saturation	3%	20-50%

**Figure 1:**
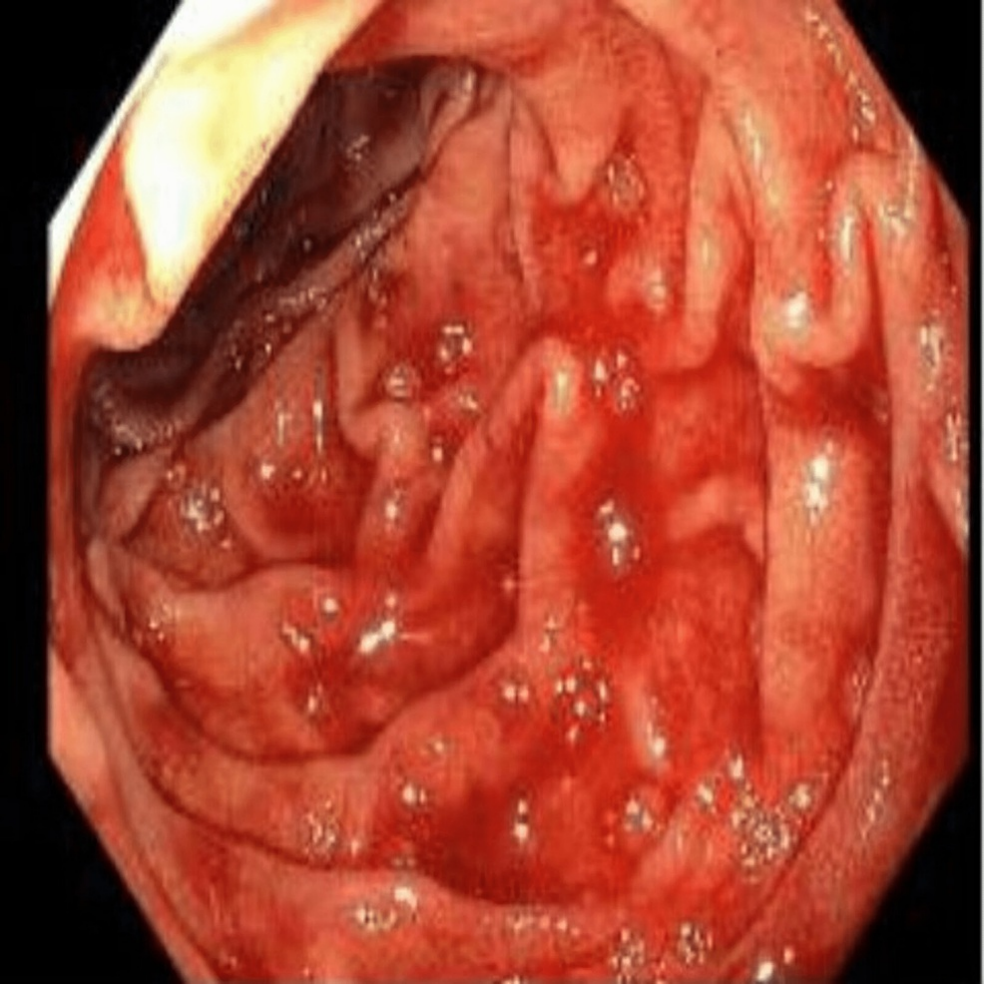
Duodenal Dieulafoy lesion in the second part of the duodenum.

Her chest X-ray showed mild pulmonary vascular congestion with bibasilar infiltrates. The patient started treatment with the antibiotics vancomycin and meropenem for pneumonia. She had worsening hypoxia, requiring the initiation of a high-flow nasal cannula and transfer to the intensive care unit. Laboratory findings showed worsening anemia to 5.8 g/dl, for which she received a blood transfusion. The CT (computed tomography) of the chest showed bilateral pleural effusion with lower lobe atelectasis, and the CT abdomen showed a 5.1 x 3.3 cm structure in the anterior segment of hepatic segment eight, concerning complex ascites versus hepatic abscess. A HIDA scan showed no evidence of cholecystitis. The patient underwent an upper esophagogastroduodenoscopy that showed a normal esophagus, erythematous mucosa in the antrum, and a bleeding duodenal dieulafoy lesion (Figure 1). 

**Figure 2:**
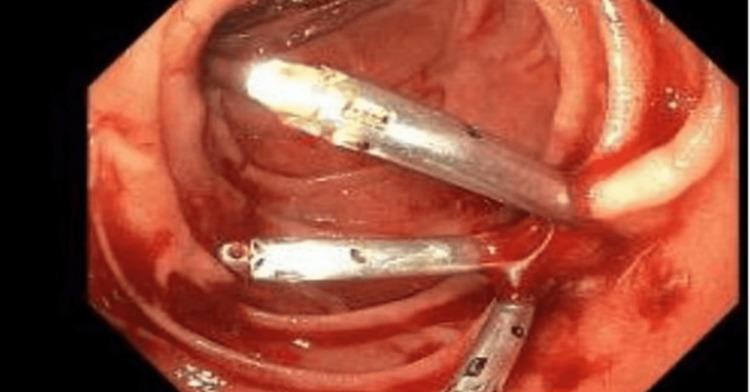
Successful clipping of the duodenal Dieulafoy lesion.

The lesion was successfully clipped using three clips, and hemostasis was achieved (Figure 2).

The patient was evaluated by surgery and was deemed not a surgical candidate for the liver abscess. She was treated with antibiotics for a liver abscess and was discharged with outpatient follow-ups. The patient is improving well; the abscess has resolved, and she has completed the course of antibiotics. 

## Discussion

Gastrointestinal bleeding can arise from various sources, and duodenal Dieulafoy lesions (DLs) represent a rare but significant cause of this condition. DLs are characterized by the presence of dilated submucosal vessels that erode the overlying mucosa, resulting in recurrent and sometimes life-threatening bleeding episodes [4]. These malformations are rare, contributing only about 1.5% of all gastrointestinal bleeding [5]. Out of that 1.5%, only one-third of these lesions are found outside of the stomach [6]. Understanding this distribution is crucial, as often, these extra-gastric sources of bleeding can be missed during endoscopic evaluations due to their relatively small size and intermittent bleeding [7].

A typical vessel supplying the gastrointestinal tract will gradually narrow as it approaches the mucosa. In the case of a Dieulafoy's lesion, however, the vessel maintains a constant diameter of 1-3mms without tapering, resulting in an abnormally enlarged vessel that eventually erodes through the mucosal surface, forming a defect through which it occasionally bleeds [8]. The large size of these vessels can be up to 10 times greater than their normal counterparts, which makes them prone to even minor mechanical injuries, resulting in bleeding [9]. This is enhanced by the pulsations of this large vessel, which disrupt the mucosal surface and expose the vessel to bowel contents, leading to further chemical erosion [10].

Another hypothesis related to the pathogenesis of DLs is related to the development of vascular steal, that is the shu mucosal erosion process [11]. Since the ischemia in this process occurs peripherally surrounding the enlarged vessel, the presence of an often visible “mucosal halo” produced by the pale, hypo-perfused mucosa may be visible [12]. Age-related gastric mucosal atrophy and arterial thrombus formation with subsequent tissue necrosis are some of the other hypotheses behind DL’s pathogenesis [13]. While no one unifying theory exists, any of these processes may lead to spontaneous rupture of the exposed vessel, which may lead to massive, potentially life-threatening hemorrhage.

While DLs can be found in all age groups, there are certain patterns that exist in patients more prone to developing these vascular malformations. By nature of age-related gastric atrophy, the elderly are at an increased risk, with males being twice as likely to develop the disease. Furthermore, while no direct link of causation has been established, there is observational data to suggest that patients who have multiple co-morbidities such as cardiovascular disease, hypertension, chronic kidney disease, diabetes, or alcohol abuse, are hospitalized, or are on NSAIDs, aspirin, and warfarin, have been observed to have these lesions more frequently [4,14,15].

The diagnosis of these lesions can be challenging. DL bleeds intermittently, and in-between bleeding episodes, even direct visualization of the affected region may not reveal the lesion. During endoscopic visualization, most commonly, DLs are seen as isolated, protruding blood vessels from the mucosa. Endoscopy is the imaging of choice in these patients, especially during acute bleeding events [16]. Hence this is an arterial malformation, active blood flow with each heartbeat will be easily visualized on endoscopy, even without the presence of a mucosal ulcer or mass effect. In the event that DL is suspected, but direct endoscopy is unable to confirm the diagnosis, endoscopic ultrasonography can be employed instead [17].

The management of duodenal DLs is similar to that of gastric DLs. Multiple approaches are efficacious in managing this vascular malformation. Endoscopically, lesions may receive epinephrine injections followed by probe coagulation or clip placement [18-20]. Endoscopic clipping was also the approach used in the case of our patient. While band ligation can also be done, it has been associated with an increased risk of bowel perforation and future bleeding [18]. Following hemostasis of duodenal DLs, successful treatment of the vessel can be confirmed using Doppler ultrasound, which should demonstrate the absence of any blood flow through the irregular artery post-procedure [21]. In the event that there is another bleeding event despite the previously described interventions, angiographic embolization, or even surgical wedge resection can be utilized [22].

Thanks to advances in endoscopy, the detection rate of DLs has increased, leading to a dramatic decrease in the mortality rate of DL bleeding from 80% to 8.6% [6]. However, this also goes to show that if unrecognized or missed, DLs can have an incredibly high death toll.

## Conclusions

In conclusion, duodenal DL is a rare but potentially life-threatening condition that leads to severe GI bleeding. Diagnosis can be challenging due to asymptomatic presentation and intermitted bleeding. Early detection of the lesions by endoscopy, angiography, and capsule endoscopy helps in prompt diagnosis, effective treatment, and the prevention of complications. Management aims to achieve homeostasis through thermal electrocoagulation, local epinephrine injections, sclerotherapy, and clipping. In cases of recurrent bleeding, arterial embolization, and surgical intervention are needed. Early intervention and a cautious approach can significantly improve the outcome and reduce morbidity and mortality.
